# Molecular characterization of the Yp11.2 region deletion in the Chinese Han population

**DOI:** 10.1007/s00414-021-02596-x

**Published:** 2021-04-26

**Authors:** Qianqian Pang, Qingai Lin, Di Wang, Zhenghao Sun, Junfang Wang

**Affiliations:** 1 Jining Medical University, Jining, 272067 China; 2 Center for Forensic Science, Jining Medical University, Jining, 272067 China

**Keywords:** Sequence-tagged site (STS) loci, Yp11.2 region, Large-scale deletion, Amelogenesis imperfecta

## Abstract

The Y chromosome is male-specific and is important for spermatogenesis and male fertility. However, the Y chromosome is poorly characterized due to massive palindromes and inverted repeats, which increase the likelihood of genomic rearrangements, resulting in short tandem repeats on the Y chromosome or long fragment deletions. The present study reports a large-scale (2.573~2.648 Mb) deletion in the Yp11.2 region in a Chinese population based on the analysis of 34 selected Y-specific sequence-tagged sites and subsequent sequencing of the breakpoint junctions on the Y chromosome from 5,068,482–5,142,391 bp to 7,715,462–7,716,695 bp. The results of sequence analysis indicated that the deleted region included part or all of the following five genes: *PCDH11Y*, *TSPY*, *AMELY*, *TBL1Y*, and *RKY*. These genes are associated with spermatogenesis or amelogenesis and various other processes; however, specific physiological functions and molecular mechanisms of these genes remain unclear. Notably, individuals with this deletion pattern did not have an obvious pathological phenotype but manifested some degree of amelogenesis imperfecta.

**Supplementary Information:**

The online version contains supplementary material available at 10.1007/s00414-021-02596-x.

## Introduction

The human genome contains many genetic variants. Two decades of the studies of human Y chromosome variability have determined a number of aspects of population histories and male-biased behaviors. The Y chromosome contains the PAR (pseudoautosomal region) and MSY (male-specific region of the Y chromosome). During meiosis, the PARs at both ends can recombine with the X chromosome, and the MSY does not recombine, resulting in paternal haploid inheritance [1]. This inheritance pattern provides for unique advantages of the use of the STR loci located in the MSY region in forensic science. The human MSY was fully sequenced in 2003 [2]; however, the complexity of the Y chromosome sequences, which are rich in segmental duplications and repeats, makes it almost impossible to accurately assemble these sequences using short-read sequencing technologies.

**Fig. 1:**
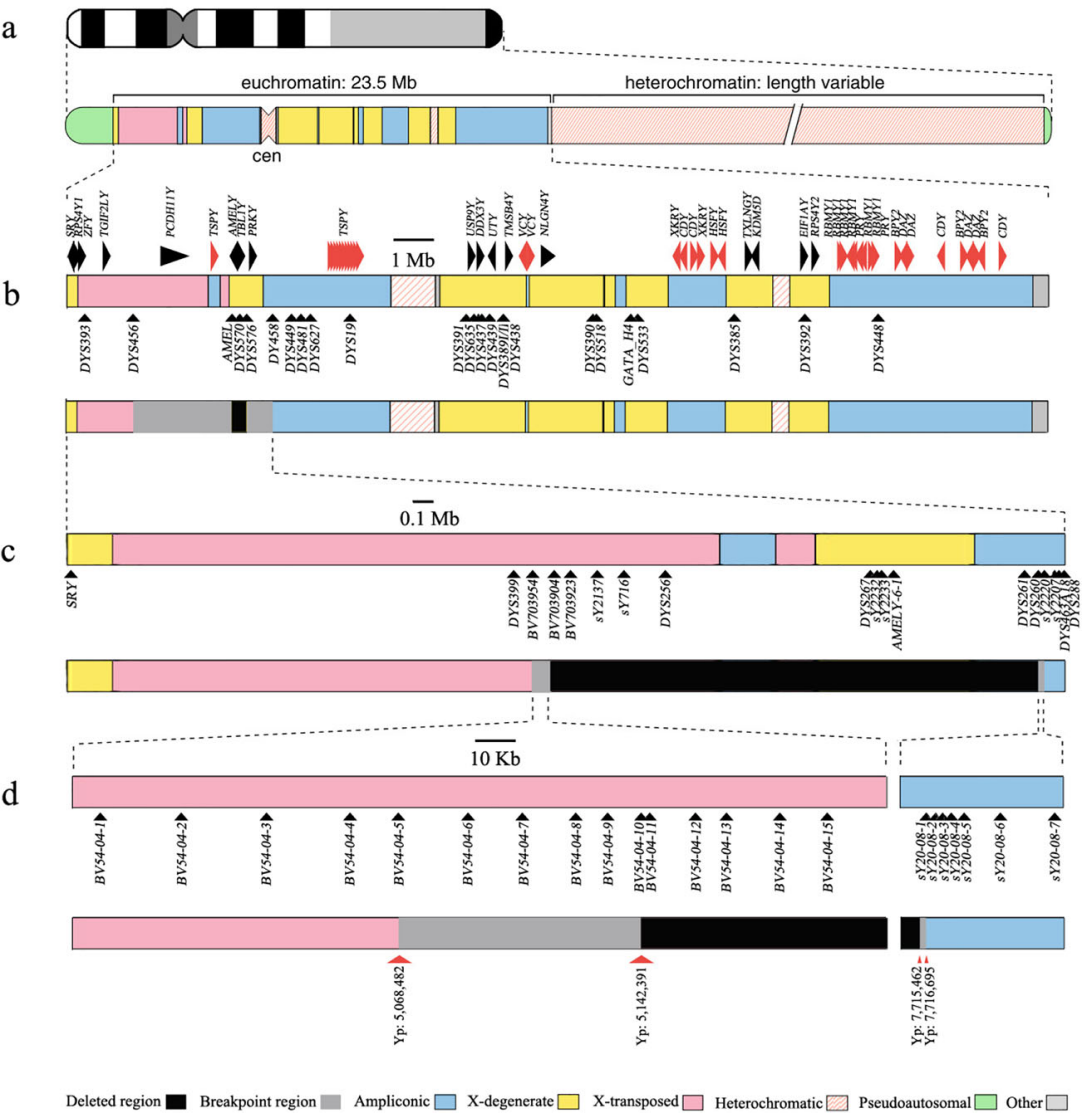
The genotyping schedule of STS loci and the deletion map in this study. **a** Ideogram of the human Y chromosome. **b** Expanded view of human MSY euchromatin showing palindromes and genes as arrowheads pointing in the direction of transcription. The location of Y-STRs in the MSY region is shown below. **c** Scheme of low-resolution mapping based on known STSs. **d** Scheme of high-resolution mapping based on newly selected loci

Human MSY sequences can be classified into the following three major classes: the X-degenerate (XDG) region with variable degrees of sequence similarity to the X chromosome; the ampliconic segment composed of sequences with high similarity to other sequences in the MSY and containing a large number of palindromes; and the X-transposed region (XTR) transferred from the long arm of the X chromosome (Fig. 1a).

The MSY contains a large number of highly homologous repetitive sequences, resulting in a highly unstable structure in this region susceptible to NAHR (nonallelic homologous recombination) within the chromosome, which leads to structural rearrangements in the Y chromosome, such as deletions, inversions, or repetitions. The MSY has a considerable impact on the forensic identification of Y-STR (STR of the Y chromosome) dependence. For example, AMELY (amelogenin Y-linked)-negative male samples can be incorrectly genotyped as females [3]. During evolution, the Y chromosome has acquired many testis-specific genes responsible for spermatogenesis [4]. Alterations in these genes are associated with several male fertility-specific traits. For example, the lack of the AZF (azoospermia factor) region can alter spermatogenesis and may result in male infertility, which is primary manifested as azoospermia and oligospermia [4].

The Y chromosome is important for spermatogenesis and male fertility and may be involved in schizophrenia and other diseases [5–7]. Moreover, males may play a larger role than females in the demic diffusion model based on the analysis of the Y chromosome [8]. The suppression of recombination causes MSY degeneration, intrachromosomal rearrangements, evolution of ampliconic repeat regions, and the accumulation of male gametogenesis genes. The loss of the Y chromosome in the peripheral blood was recently shown to be associated with increased risk for all-cause mortality and diseases, such as various forms of cancer, Alzheimer’s disease, and other conditions in aging men.

The frequencies of the deletions of Y-STRs located in the MSY region, such as *DYS393* and *DYS19*, have been reported to increase in various populations; thus, the types of identified deletions in the Yp11.2 region have been gradually diversified [9–11]. However, Y-STRs are scattered on the Y chromosome, and the intermediate space is large. Deletions of the corresponding regions of the Y chromosome represented by invalid amplification of one or several Y-STRs are complex and diverse. The detection of other genetic markers in these regions is required to detect the presence of the STS sites and to define the deletions of these regions of the Y chromosome in detail [12].

The present Yp11.2 deletion study was initiated based on the observation of *Amelogenin Y* and Y-STR (*AMELY*-*DYS570*-*DYS576*) null alleles in China in forensic studies. The present study identified 34 STS loci that can be used for the detection of Yp11.2 region deletions by screening the STS sites in the Yp11.2 region. The data obtained based on these STSs were used to accurately determine the location of the deletion junction in the Yp11.2 region of two Chinese males from Yp 5,068,482–5,142,391 bp to Yp 7,715,462–7,716,695 bp.

## Materials and methods

### Samples and DNA extraction

Yp11.2 deletion, positive, and negative samples were provided by the center for forensic science of Jining Medical University. DNA was extracted by a genomic DNA extraction kit (Tiangen Biotech, China) from fresh peripheral blood according to the manufacturer’s instructions.

### STS loci mapping

Locations of the SRY and STS loci in the Y chromosome were obtained from the UCSC Genome database (http://genome.ucsc.edu/cgi-bin/hgTracks), and the sequence of the Y chromosome was obtained from the NCBI database (https://www.ncbi.nlm.nih.gov/nuccore/NC_000024.10?from=2686000&to=7912000&report=genbank&strand=true). The primers were designed by using Lasergene and BLAST on the NCBI website. The primer sequences are shown in Table 1.

**Table 1:** Information of STS loci primers

Markers	Sequence	Product size (bp)	Annealing temperature (°C)
*SRY-F*	ACTGGTATCCCAGCTGCTTGC	228	62
*SRY-R*	AAGAGAATATTCCCGCTCTCCGG		
*DYS399-F*	CTGTAAACAAGATTGGGC	326	57
*DYS399-R*	TCCATTTACTTAAAATGG		
*BV703954-F*	GGCTCCTTGTTGCCTATGC	352	60
*BV703954-R*	ACTGGAGCAAAGGTGACTCTTGC		
*BV703904-F*	GTTAATGTTGGTAGACATCAATGCAGCTTAG	401	62
*BV703904-R*	CTTGTAAATGTTGTCCTTGGGCTTGC		
*BV703923-F*	GCCTATCTCTTAGCTGATTATCTCCTG	379	62
*BV703923-R*	ACTCTCCTTAAGTTCCACAGGAGATC		
*sY2137-F*	CTTGCTGATGTCCACCGTCTTG	261	62
*sY2137-R*	TGAATAGATCCCCCCAACCCAAATTC		
*sY716-F*	TTCACACAGTGGCTGAATTACTTTACATTCC	307	60
*sY716-R*	CTCATAAGAAGACATACAGGTAGCC		
*DYS256-F*	GGCTTTGCTAGGTAAGACCCAC	303	62
*DYS256-R*	AAGCACGCCTACCTTCACCTG		
*DYS267-F*	CATGAGGAGGCATTCTGACTCTTAG	243	62
*DYS267-R*	AAGGCTGCTGTGGCCAGACT		
*sY2232-F*	ACGTGTGTGGCTAGACGATCAGA	255	62
*sY2232-R*	CCAATCTCCTTAGCCTGATATAGGAG		
*sY2233-F*	TGTAGCCCAGGAGGCTCTCAAT	148	62
*sY2233-R*	GAGACACATCAACTCACAGCTGAC		
*DYS261-F*	CTGGACTGCACAAAACAACA	293	57
*DYS261-R*	AGAATATGGTGGGTGGGACT		
*DYS260-F*	ACTAAAACACCATTAGAAACAAAGG	309	57
*DYS260-R*	CTGAGCAACATAGTGACCCC		
*sY2220-F*	CATAGGAAGGGCAGTGCTTG	77	57
*sY2220-R*	CCATCAGAGAAGCAAGGAGG		
*sY2207-F*	ATCCTTCCCATCTCAGCTCCAG	269	62
*sY2207-R*	TCCAGAACTGACTCTAGAGCAAGG		
*DYS463A18-F*	TGATGTAGACTAAGAGCCACAGAGCTT	199	62
*DYS463A18-R*	GCATGAGGTTGTGTGACTTGACTG		
*DYS288-F*	GCTTTGCTTGTCATTTCAGAGAATCACACA	124	62
*DYS288-R*	GCAGTCTCATTACAAATACCTGGACACT		
*BV54-04-1*	CTGTGTCAACGTGTGTTCATGTCAAC	690	62
*BV54-04-1*	CACTGCATTCTCATTATCAAACAGCACC		
*BV54-04-2*	GCTCCTCACAGTGACACGTG	326	62
*BV54-04-2*	GGCACTTCACTTATCCAGTATCACC		
*BV54-04-3*	CTCCATGCTCAATTAGTCCCTAGAGT	441	62
*BV54-04-3*	CAACTATAGTGGGAAGCAGCATAGTG		
*BV54-04-4*	GTGTCTCCATGCCTTGTTACACAG	433	62
*BV54-04-4*	GTACAGGTCAAACCATTTGTCCACTG		
*BV54-04-5*	CACTATCAACATTTTGGTCAAAGTCATTCAGC	377	62
*BV54-04-5*	ACTATCTCAGGACAGTCAGAATGACGATT		
*BV54-04-6*	GAGATCTTGACCTGTGAATTTTAGTTCCTTG	527	62
*BV54-04-6*	ATCGTATCATTCTTATGCCTTTGCGTCC		
*BV54-04-7*	CCAAAGAAACTGCTGAGTTATCTTCCAG	286	62
*BV54-04-7*	GCAGCACTGTGTGGAATTCTGTTGTTAA		
*BV54-04-8*	CCATTCACTACTCATATGTCTCATGTAGTTAG	441	62
*BV54-04-8*	CAGTGAAGGGGAGATGTTTATAGGAG		
*BV54-04-9*	CCTCGGAAATCGATATATCACATCAGTG	359	62
*BV54-04-10*	AGCAAGCCTAAAGCAGGTGTTACAAAAC		
*BV54-04-10*	GCTGGGTTCATTGCCTAGAGTCT	319	62
*BV54-04-10*	CTCCTCATATCATCCAATGTTTAGGTGC		
*BV54-04-11*	CACAGAATAGCATTTAGGATGGTGTAGAC	305	62
*BV54-04-11*	GGGCTATCTCTCATAGAAACAAACAGC		
*BV54-04-12*	GTGGTGGTAAAATGCTGTCCATGTG	347	62
*BV54-04-12*	CTCACTTTTGATGGTCACAAGTTGTACCTC		
*BV54-04-13*	GCTTAATGTCCTCATGCAAATATGACTTTTATAGC	304	62
*BV54-04-13*	GGGAAGTTGTAGCAACTTTAGATCCAAG		
*BV54-04-14*	GTCAATGCACTACAACTGCTGTTTGCTA	396	62
*BV54-04-14*	GATGACTGGAATAGTTGATGCATCAGAG		
*BV54-04-15*	GGTGCTCTCATCTAAGATTAGTTGACTCT	311	62
*BV54-04-15*	GTTGAACTGTTCTTGCACCTCAGG		
*sY20-08-1*	CAGACAGCCTGACATGCATAGTGAAT	452	62
*sY20-08-1*	AGCATTGTGTCTATCAGCTATTGCTCC		
*sY20-08-2*	GACTGGACTAGATTGACTACGCTG	447	62
*sY20-08-2*	CCCTATCTGTTCATTTAATTGGCACTGC		
*sY20-08-3*	GGAGAGCTGACATTAATAGTGCTATTCC	372	62
*sY20-08-3*	CTCTCTAGCAGTCAACCAGGTATC		
*sY20-08-4*	GCCTATCTTTGGAAGTGGCCACTT	512	62
*sY20-08-4*	GCACTTATTCACTGGCATCATCTCC		
*sY20-08-5*	GCTGCAGTGTCAGATCTGGG	257	62
*sY20-08-5*	CACTGCCCTTCCTATGCTGC		
*sY20-08-6*	GACACACATGTGGACTTGTATTCATGCC	389	62
*sY20-08-6*	GCAGCCTCTACCAAGCCTTTG		
*sY20-08-7*	CGGTCTATGGGCCTTAGATCC	269	62
*sY20-08-7*	TGAGGCAGGAAGCAGTGACAG		

Gradient PCR was used to determine suitable annealing temperature for each primer pair. Annealing temperatures are shown in Table 1. Genomic DNA extracted from the blood sample was used as a template for PCR amplification. PCR amplification was carried out using 10 μL of 2×Taq PCR Master Mix II (TIANGEN, China), 0.5 μL of each of the primers (10 μmol/L), 2 μL of DNA template (200~400 ng/μL), and 7 μL of nuclease-free water in a total volume of 20 μL. Thermal cycling conditions were as follows: 95 °C for 5 min, 32 cycles at 94 °C for 30 s, annealing temperature for 30 s, and 72 °C for 40 s, and a final extension at 72 °C for 10 min. Amplified PCR products were separated by 1.0% agarose gel electrophoresis and visualized under a UV light source.

### Long PCR amplification

Long PCR amplification was performed using a KOD FX Neo kit (Toyobo Life Science, Japan) according to the manufacturer’s instruction. The samples contained 10 μL of 2× PCR buffer for KOD FX Neo, 0.5 μL of each of the primers (10 μmol/L), 0.5 μL of dNTP (2 mM each), 1 μL of KOD FX Neo (1.0 unit/μL), 2 μL of DNA template (200~400 ng/μL), and 4 μL of nuclease-free water in a total volume of 20 μL. The thermal cycling conditions were as follows: 95 °C for 5 min, 32 cycles at 94 °C for 30 s, 62 °C for 30 s, and 72 °C for 40 min, and a final extension at 72 °C for 1 h. Amplified PCR products were separated by 0.7% agarose gel electrophoresis and visualized under a UV light source.

## Results

Two samples from paternity testing were from males, and all autosomal STRs were successfully genotyped; we were unable to detect *AMELY* by two different amplification kits. The results of Y-STR genotyping showed that *DYS570* and *DYS576* were missing; however, *DYS456*, *DYS458*, and other Y-STRs were successfully genotyped (Fig. 1b). Therefore, the breakpoint junctions were identified as *DYS456*-*AMELY* and *DYS576*-*DYS458*.

Sixteen known Y chromosome-specific STS loci located in these two breakpoint regions were used for low-resolution mapping (Fig. 1c). Six STS loci, comprising *DYS399*, *BV703954*, *sY2207*, *sY2220*, *DYS463A18*, and *DYS288*, were present, and ten loci (*BV703904*, *BV703923*, *sY2137*, *sY716*, *DYS256*, *DYS267*, *sY2232*, *sY2233*, *DYS261*, and *DYS260*) were deleted in both males (Fig. 1c, top). The results of the STS loci amplification are shown in Table 2.

**Table 2:** Detection of STS loci in two male samples

Markers	Sample 1	Sample 2	Position (Mb)
*SRY*	+	+	2.787
*DYS399*	+	+	4.988
*BV703954*	+	+	5.080
*BV703904*	-	-	5.193
*BV703923*	-	-	5.274
*sY2137*	-	-	5.420
*sY716*	-	-	5.556
*DYS256*	-	-	5.747
*DYS267*	-	-	6.758
*sY2232*	-	-	6.788
*sY2233*	-	-	6.789
*DYS261*	-	-	7.554
*DYS260*	-	-	7.715
*sY2220*	+	+	7.726
*sY2207*	+	+	7.749
*DYS463A18*	+	+	7.775
*DYS288*	+	+	7.790
*BV54-04-1*	+	+	5.007
*BV54-04-2*	+	+	5.027
*BV54-04-3*	+	+	5.048
*BV54-04-4*	+	+	5.068
*BV54-04-5*	+	+	5.080
*BV54-04-6*	/	/	5.097
*BV54-04-7*	/	/	5.111
*BV54-04-8*	/	/	5.124
*BV54-04-9*	/	/	5.132
*BV54-04-10*	-	-	5.140
*BV54-04-11*	-	-	5.142
*BV54-04-12*	-	-	5.153
*BV54-04-13*	-	-	5.161
*BV54-04-14*	-	-	5.174
*BV54-04-15*	-	-	5.186
*sY20-08-1*	+	+	7.717
*sY20-08-2*	+	+	7.719
*sY20-08-3*	+	+	7.721
*sY20-08-4*	+	+	7.723
*sY20-08-5*	+	+	7.726
*sY20-08-6*	+	+	7.734
*sY20-08-7*	+	+	7.748

“+”: the locus was existed on the position. “-”: the locus was absent on the position. “/”: the product was not Y-specific on the position

The data indicated that the distal breakpoint was located between *BV703954* (5.080 Mb) and *BV703904* (5.193 Mb). The proximal breakpoint was located between *DYS260* (7.715 Mb) and *sY2220* (7.726 Mb) (Fig. 1c, bottom); additional closely spaced STSs (~20 kb) were used for high-resolution mapping of the breakpoint junctions. The distal *BV54-04-1*, *BV54-04-2*, *BV54-04-3*, *BV54-04-4*, and *BV54-04-5* breakpoints were positive, and the *BV54-04-10*, *BV54-04-11*, *BV54-04-12*, *BV54-04-13*, *BV54-04-14*, and *BV54-04-15* breakpoints were negative. We were unable to determine the presence of the *BV54-04-6*, *BV54-04-7*, *BV54-04-8*, and *BV54-04-9* loci due to poor specificity of the binding on the Y chromosome. The proximal *sY20-08-1*, *sY20-08-2*, *sY20-08-3*, *sY20-08-4*, *sY20-08-5*, *sY20-08-6*, and *sY20-08-7* loci were positive.

Finally, *BV54-04-5*, *BV54-04-6*, *BV54-04-7*, *BV54-04-8*, and *BV54-04-9* were used as forward primers and *sY20-08-1* was used as a reverse primer for long PCR amplification to detect the deletion junction sequences; unfortunately, all results of the long PCR amplifications were negative.

## Discussion

The nulls of the AMELY and Y-STR loci in the Yp11.2 region have been frequently reported in the Chinese population. The present study demonstrated that the Y-STRs of two Chinese males contained *DYS570* and *DYS576* combined with the AMELY deletion, and other Y-STRs were normally amplified. We used a three-stage strategy of combined low-resolution STS mapping, high-resolution STS mapping, and long PCR amplification [8]. A total of 34 STSs between *DYS456* and *DYS458* (*DYS399*, *BV703954*, *BV54-04-1*, *BV54-04-2*, B*V54-04-3*, *BV54-04-4*, *BV54-04-5*, *BV54-04-10*, *BV54-04-11*, *BV54-04-12*, *BV54-04-13*, *BV54-04-14*, *BV54-04-15*, *BV703904*, *BV703923*, *sY2137*, *sY716*, *DYS256*, *DYS267*, *sY2232*, *sY2233*, *DYS261*, *DYS260*, *sY20-08-1*, *sY20-08-2*, *sY20-08-3*, *sY20-08-4*, *sY20-08-5*, *sY20-08-6*, *sY20-08-7*, *sY2207*, *sY2220*, *DYS463A18*, and *DYS288*) were mapped to this region. The deletion junctions of the two Chinese males we tested based on these STS loci starting from 5,068,482–5,142,391 to 7,715,462–7,716,695, and the size of the deletion region was 2.573~2.648 Mb. The breakpoint regions were too long (>40 kb) or/and complex to amplify; thus, long PCR amplification did not obtain any long fragments, and all results were negative, suggesting that the length of the missing fragments was close to 2.573 Mb.

The design of STSs for high-resolution mapping in the breakpoint region is difficult because this region has extremely high homology with the X, 3, and 5 chromosomes; thus, it was not possible to precisely locate the deletion site.

Sequence analysis indicated that the missing region contained *PCDH11Y* (protocadherin 11 Y-linked), *TSPY* (testis-specific protein Y-linked 1), *AMELY*, *TBL1Y* (transducin beta-like 1 Y-linked), and *PRKY* (protein kinase Y-linked). *PCDH11Y* plays a role in cell-cell recognition during central nervous system development, belongs to the protocadherin family, and is very closely related to its paralog on the X chromosome. *PCDH11Y* is essential for spermatogonial differentiation and initiation of meiosis [13] and may also be a candidate marker for susceptibility to psychiatric disorders [14]. *TSPY* is expressed only in testicular tissue and may be involved in spermatogenesis. Previous studies demonstrated that *TSPY1* has physiological functions in the proliferation and differentiation of spermatogonia during spermatogenesis [15]. Remarkably, *TSPY1* is also involved in the initiation and development of many tumors by activating the PI3K/AKT and RAS signaling pathways by suppressing IGFBP3 expression [16]. *Amelogenins* are useful for sex identification and are involved in biomineralization during tooth enamel development [17]. Mutations in a related gene on chromosome X cause X-linked amelogenesis imperfecta. *Amelogenin* can also be used to monitor bone marrow implantation in patients with sex-mismatched bone marrow transplantation. *TBL1Y* is a Y-linked homolog of *TBL1X* that was considered a novel candidate for hereditary hearing loss acting via the Wnt signaling pathway [18]. The expression pattern and function of *TBL1Y* are unknown; however, this gene may play some other roles in maleness due to specific chromatin binding and transcription corepressor activity. *PRKY* is similar to the protein kinase X-linked gene in the PAR and is classified as a transcribed pseudogene. However, abnormal recombination between this gene and a related gene on the chromosome X is a frequent cause of XX males and XY females [19]. Meyfour et al. reported alterations in the mRNA and protein levels of *TBL1Y*, *PCDH11Y*, *ZFY*, *KDM5D*, *USP9Y*, *RPS4Y1*, *DDX3Y*, *PRY*, *XKRY*, *BCORP1*, *RBMY*, *HSFY*, and *UTY* accompanied by changes in intracellular localization during cardiac differentiation of human embryonic stem cells (hESCs) [20]. In some cases, repeated mutations of the *AMELY*, *TBL1Y*, and *PRKY* regions have been detected without obvious deformities [21].

The results of the present study suggest that the C-terminus could have been deleted from the full-length *PCDH11Y*, even though two males did not show any symptoms of infertility or mental illness. The Y chromosome contains numerous *TSPY* repeats. Changes in the copy number of *TSPY* can change the tumorigenic ability of prostate cancer cells in nude mice and may lead to prostate cancer in men. However, deletion of a single copy of *TSPY* did not result in a phenotype in the present study. Family investigation showed that males of this family generally have poor teeth conditions, such as amelogenesis imperfecta (Fig. S1), with no obvious pathological manifestations. In addition, the effects of this deletion on physiological conditions of the two male subjects cannot be confirmed without systematic health examinations.

The effects of the gene and protein functions on human physiology and pathology require comparative studies. Male infertility is related not only to a certain gene or region in the Y chromosome. Many autosomal proteins loss-of-function mutations of autosomal proteins can also cause male infertility, including the function of the Golgi matrix protein GM130 in male-specific germ cells [22]. Knockout of *GM130* resulted in the absence of acrosomes in mice and caused male infertility [23]. Analysis of the Yp11.2 region deletion and individual phenotypes can provide information for future basic studies on male infertility and other related diseases, such as enamel hypoplasia and deafness, and the data can be used in the studies of gene expression and functions of the corresponding proteins.

The majority of the studies on Y chromosome deletions related to male infertility involved the AZF region; however, the relationship between other regions outside the AZF region and male infertility is unknown. The lack of data in these areas is mainly due to the lack of convenient and fast commercial kits. We can optimize the STS primer system used in the present study to construct a multiple amplification system, which can be used for specific amplification of the Yp11.2 region and will be suitable for large-scale screening. Moreover, the present study provides theoretical data and technical support for investigations of the Y chromosome deletions in Chinese men. The system used in the present study is suitable for rapid clinical diagnostic and genetic screening of infertility in men.

The missing regions and related pathological phenotype reported in this study have been determined. The Yp11.2 deletion region identified in the present study can be used as a negative reference to analyze whether the cause of the infertility is associated with microdeletions in the Y chromosome, to exclude the genetic cause and thus confirm the actual pathogenesis of male infertility, and to provide a more accurate reference for the targeted and special treatments. Moreover, identification of the cause of infertility will facilitate early diagnosis and treatment of reproductive disorders in the offspring.

The present study detected 38 STSs in the Yp11.2 region between *DYS456* and *DYS458*, confirming the deletion junction in the two Chinese males from 5,068,482–5,142,391 to 7,715,462–7,716,695, and the size of the deletion region was 2.573~2.648 Mb. A total of 34 chromosome Y-specific STSs were confirmed to be suitable for the deletion mapping in this region. Molecular analysis demonstrated that the missing region contained five genes: *PCDH11Y*, *TSPY*, *AMELY*, *TBL1Y*, and *PRKY*. Two males presented with certain signs of amelogenesis imperfecta with no obvious pathological manifestations. The data of the present study can provide theoretical and technical support for the investigations of gene functions and Yp11.2 region deletion mapping and for the development of multiple amplification systems.

## Supplementary Information

ESM 1 (PNG 2556 kb)

High resolution image (EPS 24212 kb)

## Abbreviations

AMEL: Amelogenin
AMELX: Amelogenin X-linked
AMELY: Amelogenin Y-linked
AZF: Azoospermia factor
MSY: Male-specific region of Y chromosome
PAR: Pseudoautosomal region
PCDH11Y: Protocadherin 11 Y-linked
PRKY: Protein kinase Y-linked
SRY: Sex-determining region Y
STR: Short tandem repeat
STS: Sequence-tagged site
TBL1Y: Transducin beta-like 1 Y-linked
TSPY: Testis-specific protein Y-linked
Y-STR: Y chromosome STR

## Declarations

Bloodstains and fresh peripheral blood samples were acquired from individuals in the Chinese Han population of Jining City. All procedures performed in the present study were approved by the Medical Ethics Committee of Jining Medical School in accordance with the 2013 Helsinki Declaration.
