# Supplementary figures and images for: Molecular characterization of the Yp11.2 region deletion in the Chinese Han population
Source: Int J Legal Med. 2021 Apr 26;135(4):1351–8. doi: 10.1007/s00414-021-02596-x (PMC8205872; doi:10.1007/s00414-021-02596-x)

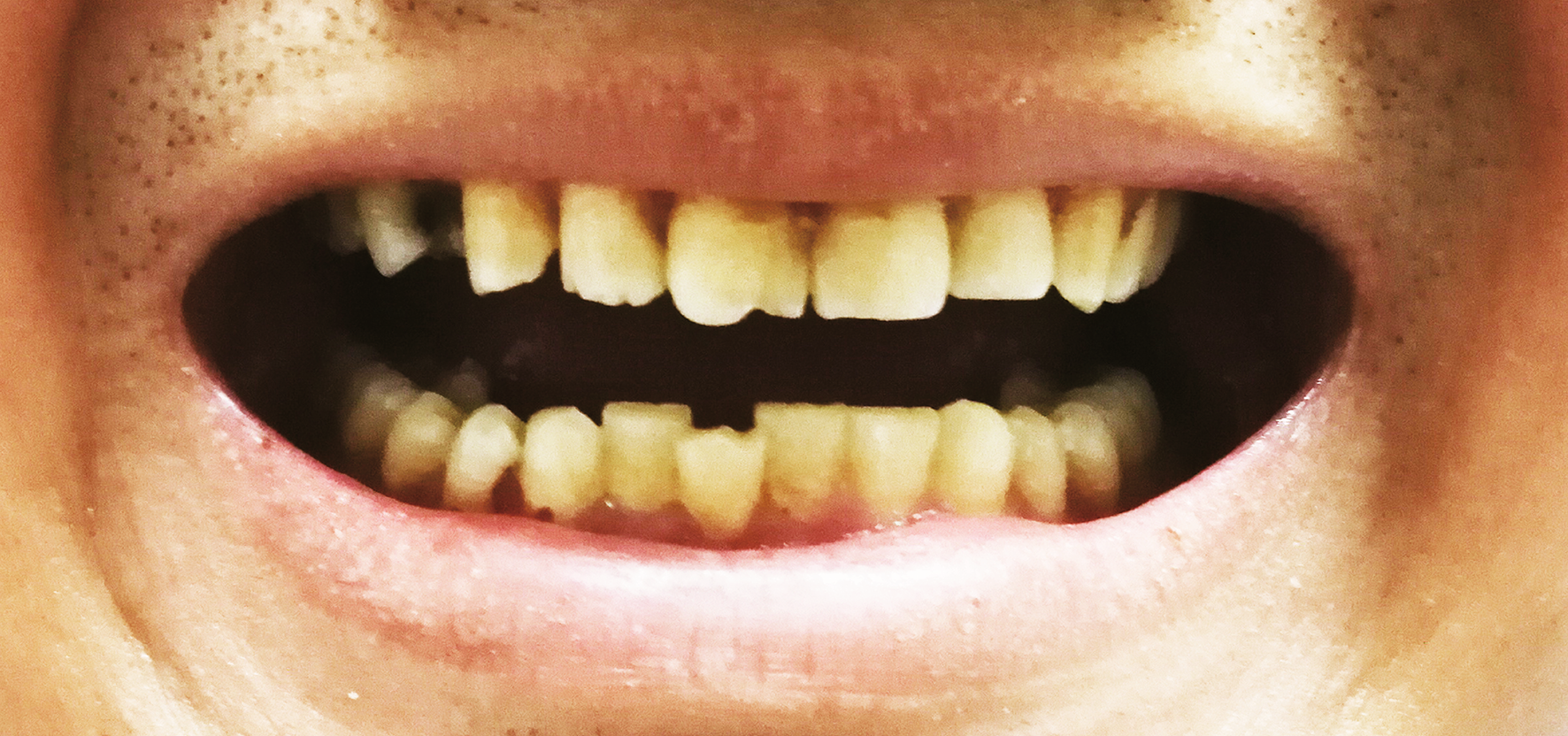

Supplement: Supplementary file 1 — (PNG 2556 kb) [file 414_2021_2596_Fig2_ESM.png]
